# Macroscopic inelastic scattering imaging using a hyperspectral line-scanning system identifies invasive breast cancer in lumpectomy and mastectomy specimens

**DOI:** 10.1117/1.JBO.29.6.065004

**Published:** 2024-06-06

**Authors:** Sandryne David, Hugo Tavera, Tran Trang, Frédérick Dallaire, François Daoust, Francine Tremblay, Lara Richer, Sarkis Meterissian, Frédéric Leblond

**Affiliations:** a Polytechnique Montréal, Department of Engineering Physics, Montreal, Quebec, Canada; b Centre de recherche du Centre hospitalier de l’Université de Montréal (CRCHUM), Montreal, Quebec, Canada; c McGill University Health Center (MUHC), Department of Surgery, Montreal, Quebec, Canada; d McGill University Health Center (MUHC), Department of Pathology, Montreal, Quebec, Canada; e Institut du cancer de Montréal, Montreal, Quebec, Canada

**Keywords:** Raman spectroscopy, breast cancer, breast-conserving surgery, machine learning, biomedical imaging, tissue optics, biochemistry, support vector machines

## Abstract

**Significance:**

Of patients with early-stage breast cancer, 60% to 75% undergo breast-conserving surgery. Of those, 20% or more need a second surgery because of an incomplete tumor resection only discovered days after surgery. An intraoperative imaging technology allowing cancer detection on the margins of breast specimens could reduce re-excision procedure rates and improve patient survival.

**Aim:**

We aimed to develop an experimental protocol using hyperspectral line-scanning Raman spectroscopy to image fresh breast specimens from cancer patients. Our objective was to determine whether macroscopic specimen images could be produced to distinguish invasive breast cancer from normal tissue structures.

**Approach:**

A hyperspectral inelastic scattering imaging instrument was used to interrogate eight specimens from six patients undergoing breast cancer surgery. Machine learning models trained with a different system to distinguish cancer from normal breast structures were used to produce tissue maps with a field-of-view of $1\;{\mathrm{cm}}^{2}$ classifying each pixel as either cancer, adipose, or other normal tissues. The predictive model results were compared with spatially correlated histology maps of the specimens.

**Results:**

A total of eight specimens from six patients were imaged. Four of the hyperspectral images were associated with specimens containing cancer cells that were correctly identified by the new *ex vivo* pathology technique. The images associated with the remaining four specimens had no histologically detectable cancer cells, and this was also correctly predicted by the instrument.

**Conclusions:**

We showed the potential of hyperspectral Raman imaging as an intraoperative breast cancer margin assessment technique that could help surgeons improve cosmesis and reduce the number of repeat procedures in breast cancer surgery.

## 1 Introduction

Breast cancer is the most diagnosed cancer worldwide and the deadliest cancer in women.^1^ The most common breast cancer types are ductal carcinoma and lobular carcinoma. Ductal carcinoma originates in epithelial cells of the ducts carrying milk in the breast, whereas lobular carcinoma originates from cells of the lobules that constitute the glands producing milk. Both of these carcinomas can be *in situ* or invasive.^2^ *In situ* cancers are still contained in their structure of origin, whereas invasive cancer cells have spread into surrounding tissues.^2^ Invasive ductal carcinoma and invasive lobular carcinoma represent 70% to 80%^3^ and 5% to 15%^4^^,^^5^ of invasive breast cancers, respectively. Mucinous carcinoma is a rare type of invasive breast cancer that accounts for 2% of all breast carcinoma. It is characterized by neoplastic epithelial cells floating within extracellular mucin.^6^ Other less frequent breast cancer types include tubular carcinoma (1% to 2% of invasive breast cancers), medullary carcinoma (less than 5% of all invasive breast cancers),^8^ papillary carcinoma (0.5% of all breast cancers),^9^ and micropapillary carcinoma (0.9% to 2% of all breast cancers).^10^^,^^11^ Regardless of the cancer histotype, the standard of care involves surgical excision of the tumor followed by radiotherapy. Surgical options include breast-conserving surgery (also known as lumpectomy), mastectomy (complete ablation of the breast), and the resection of lymph nodes. Breast-conserving surgery presents the advantage of minimizing the change in appearance of the breast and the risk of complications while having the same long-time survival rate as mastectomy for patients having a tumor under 2 cm (the largest dimension of the tumor).^12^^–^^14^ This explains why as many as 60% to 75% of patients chose to undergo breast-conserving surgery.^15^ The success of breast-conserving surgery is defined as the complete removal of the lesional breast tissue with a surrounding margin of normal tissue.^16^ Since a positive margin (i.e., cancer cells on the surface of the surgical specimen) is associated with a higher risk of cancer recurrence, extended tissue resection is required when positive margins are detected.^17^ This is important because 20% or more of all breast-conserving surgery procedures result in margins with residual disease.^18^^–^^21^ Because the definitive margin status is only assessed postoperatively, patients with positive margins require a second operation, which leads to additional costs, patient anxiety, and an increased risk of post-surgical complications. In the United States alone, there are 26,550 re-excisions annually, costing approximately $125M.^22^

A limited number of methods were tested for *in vivo* breast tissue assessment. Ultrasound imaging was used intraoperatively but is limited in sensitivity due to a lack of molecular specificity.^17^^,^^23^ The MarginProbe from Dune Medical, which relies on electrical impedance measurements, is an *in vivo* imaging method that was approved by the Food and Drug Administration (FDA). However, both its sensitivity and specificity are low at around 70%.^24^ Fluorescence imaging was also tested *in vivo* with indocyanine green, resulting in a sensitivity and specificity of 100% and 60%, respectively.^25^ The use of LUM015 (Lumicell, Newton, Massachusetts, United States), a protease-activatable fluorescent agent, resulted in a cancer detection sensitivity and specificity of 100% and 73%, respectively.^26^ It was approved by the FDA for lumpectomy specimen imaging in imaging. Moreover, the use of the fluorescent molecular marker pegulicianine resulted in a sensitivity and specificity of 85% and 49%, respectively, in a fluorescence-guidance surgery study.^27^

To increase the sensitivity and molecular specificity of breast cancer detection, optical methods have also been developed for *ex vivo* specimen interrogation. Whole-specimen imaging techniques included photoacoustics, acousto-optics, spatial frequency domain imaging,^28^^,^^29^ X-ray,^30^ and fluorescence imaging.^31^^,^^32^ Single-point and microscopic optical imaging methods included elastic scattering and diffuse reflectance spectroscopy,^33^ bio-impedance spectroscopy,^34^ microscopy with ultraviolet surface excitation,^35^ light-sheet microscopy,^36^ nonlinear microscopy,^37^^–^^39^ optical coherence tomography,^40^ and Raman spectroscopy (RS).^41^^–^^45^ However, these methods were sometimes limited by prohibitive imaging times.

Our group previously published a paper on the use of an intraoperative RS point probe system to detect invasive breast cancer *ex vivo*.^46^ The system was used to interrogate fresh surgical specimens from 20 patients undergoing breast-conserving surgery, mastectomy, or breast reduction surgery. This resulted in 238 measurements that were spatially registered with standard histology, classifying tissue as *cancer*, *normal*, or *fat*. A technique based on support vector machines (SVMs) led to the development of predictive models, and their performance was quantified using a receiver operating characteristic (ROC) analysis. The technology was able to detect invasive breast cancer with a sensitivity of 93% and a specificity of 95%, showing the feasibility of cancer detection using RS. The classification model used a limited number of spectral features, emphasizing the importance of the protein band at $940\;{\mathrm{cm}}^{-1}$ and the phenylalanine band at $1004\;{\mathrm{cm}}^{-1}$ for breast cancer detection.

However, RS technologies have several hurdles to overcome before being integrated for real-world use to guide breast-conserving surgery. In particular, the field of view (FOV) of the point probe system was limited to 500  μm, which is incompatible with specimen margin assessment within clinically relevant timeframes. Our research group then developed a hyperspectral Raman imaging system to address this issue. The instrument had a spectral domain and a spectral resolution that were similar to the probe, with an FOV in the form of a square with sides of 1 cm. The system was a line-scanning device collecting hyperspectral data over 42×40  pixels, resulting in 1680 spectra per image. Our group demonstrated the system’s ability to detect margins among different tissue types in biological material, showing its potential for intraoperative machine learning-based molecular tissue margin characterization.^47^^,^^48^ The system was flexible and could either be used *in vivo* live during surgery or *ex vivo* for specimen analyses.

This work presents the results of a study that led to the acquisition of *ex vivo* Raman imaging datasets of human breast specimens, including normal tissue and invasive breast cancer. The dataset was acquired with the hyperspectral line-scanning Raman system. This study used the machine learning classification models developed in the previous study with the single-point probe to detect invasive breast carcinoma. The point probe models were directly applied to each pixel of the imaging system to form images. This study assessed whether Raman hyperspectral imaging could detect specific biomolecular features of breast cancer and whether the approach had potential for surgical guidance in breast-conserving surgery to reduce re-excision procedure rates.

## 2 Methods

### 2.1 RS System

Measurements were made using a macroscopic RS imaging system for which details were provided elsewhere.^48^ Briefly, the excitation branch of the Raman line-scanning system was composed of a laser centered at 785 nm providing a line-shaped laser excitation and a white light source with illumination between 400 and 700 nm. The laser and white light sources were conveyed through an excitation fiber bundle onto the sample. The Raman signal and the reflected white light were collected by the collection fiber bundle and separated by a dichroic mirror through a galvanometer-based system designed for scanning the tissue surface. An imaging spectrometer generated hyperspectral Raman images, and an RGB (red-green-blue) camera was used to collect white light images. The spatial resolution of the hyperspectral images was 250  μm, and the spectral resolution was $6\;{\mathrm{cm}}^{-1}$. The Raman spectra were acquired in the fingerprint region from 400 to $1900\;{\mathrm{cm}}^{-1}$. The measurements were made in non-contact mode at a working distance of 40 mm from the specimen. The FOV of both the Raman hyperspectral images and the white light images was $1\;{\mathrm{cm}}^{2}$. The system was controlled using custom software (LabVIEW 2018 version 18.0f2, National Instruments, Austin, Texas, United States) that allowed acquisition parameters to be set by the user, including laser power, exposure time per line, and number of repeated measurements (accumulations) for each image.^48^

### 2.2 Patient Selection

Six patients who underwent breast surgery (lumpectomy or mastectomy) following a diagnosis of invasive breast carcinoma (ductal or mucinous) were recruited for this study. All patients recruited in the study were women undergoing breast surgery for the first time. The patients did not receive neoadjuvant therapy and had a cancer grade inferior to four.

Fresh patient specimens were utilized to build an *ex vivo* dataset of Raman hyperspectral images combined with histopathological and clinical data. Informed consent was obtained before each patient underwent surgery (McGill University Health Center Ethics Committees, approval number 2021-7940). Clinical data available included age, tumor type, and size, and patient demographic details were provided (Tables 1 and 2). Each tumor size was characterized by its largest dimension measured by the pathologist at the time of intraoperative consultation.

**Table 1:** Clinical and pathological characteristics of all patients undergoing breast surgery who were recruited as part of the study.

Number of patients	6
Type of surgery	
Breast-conserving surgery	4
Mastectomy	2
Number of patients per tumor type	
Invasive ductal carcinoma	5
Invasive mucinous carcinoma	1
Tumor size average (standard deviation)	2.4 cm (0.9 cm)

**Table 2:** Demographic and clinical information of the six patients included in the study.

Patient #	Surgery type	Tumor size (cm)	Invasive cancer type	Tissue type (according to classification, after exclusion)
P1	Lumpectomy	2.5	Ductal	Fat (1421), cancer (297), and normal (245)
P2	Lumpectomy	2.2	Ductal	Fat (318), cancer (345), and normal (132)
P3	Lumpectomy	1.5	Ductal	Fat (842), cancer (2), and normal (137)
P4	Mastectomy	6.5	Mucinous	Fat (963), cancer (51), and normal (281)
P5	Lumpectomy	0.3	Ductal	Fat (1269), cancer (34), and normal (135)
P6	Skin sparing mastectomy	1.1	Ductal	Fat (1107) and normal (317)

### 2.3 Specimen Handling and *Ex Vivo* Spectroscopic Measurements

Eight specimens were extracted from six patients by a surgeon (S.M. or F.T.). The patients were labeled P1 to P6. In cases where more than one specimen was obtained for the same patient, each was labeled either S1 or S2. A total of eight samples were analyzed that were all subsequently sent to pathology for margins assessment as part of the regular clinical workflow. Prior to this, the specimens were weighed, measured, inked, and sliced into 5 mm sections, as per institutional protocols.

**Fig. 1:**
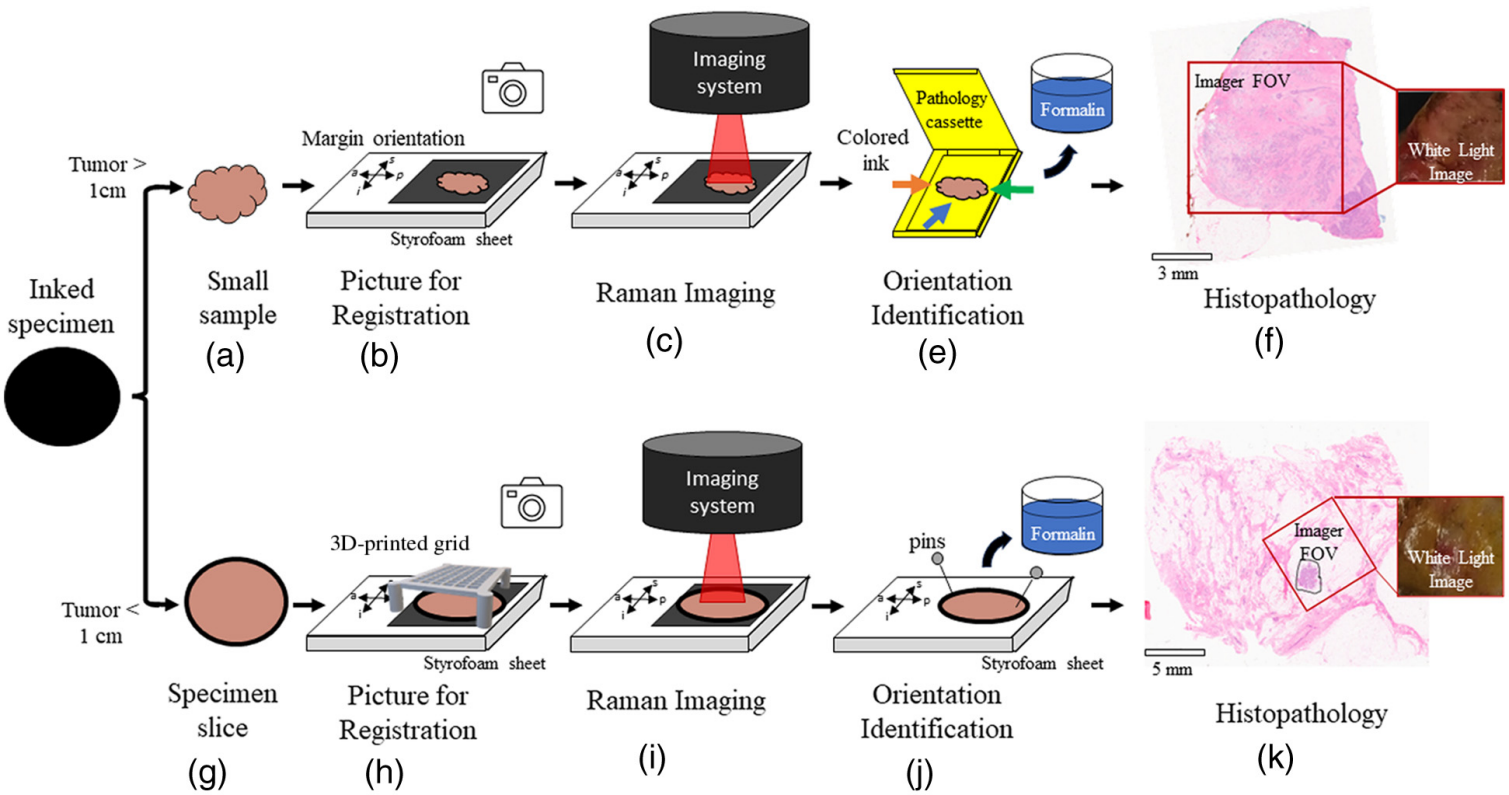
Schematic representation of the two specimen handling methods leading to *ex vivo* spectroscopic measurements: (a)–(f) tumors larger than 1 cm and (g)–(k) tumors smaller than 1 cm. (a) A sample was cut from the specimen slices and (b) placed onto a black-coated aluminum sheet disposed on an EPS sheet. The orientation of the margins was marked on the EPS sheet and a photograph was taken. (c) The EPS sheet was then placed under the Raman imaging system for measurements. (e) The sides of the sample were inked with different colors to indicate the orientation of the margins, and the sample was transferred into a pathology cassette before being put in formalin for fixation. (f) An H&E-stained image of the sample was analyzed by the pathologist. The white light image and the specimen photograph were used to register the position of the Raman measurements with the H&E image. (g) The entire specimen slice was put on a black-coated aluminum sheet (h) that was placed onto an EPS sheet. The orientation of the margins was marked on the EPS sheet, and a 3D-printed grid was placed on top without direct contact with the specimen. A photograph was taken, and the pathologist indicated a square of the grid that contained the structures of interest. (i) The EPS sheet was placed under the Raman imaging system and the grid was used to record the location of the measurement. The grid was removed without moving the specimen slice, and the Raman measurements were made. (j) The specimen slice was then transferred to another EPS sheet where it was pinned alongside the other slices of the specimen, when applicable. The orientation of the margins was indicated on the EPS sheet, and it was placed in formalin for fixation as per standard of care. (k) This resulted in an H&E-stained image of the entire specimen slice where the pathologist indicated the position of the tumor. The white light image and the specimen photograph were used to register the position of the Raman measurement on the H&E image.

Depending on the size of the tumor, Raman imaging measurements and spatial registration with histology were achieved using either of two methods (Fig. 1). All samples imaged with the system were slices of lumpectomy specimens that had a thickness of ∼5  mm. One method was used for the cases where the largest dimension of a tumor, measured *ex vivo* by the pathologist, was superior to 1 cm. For those cases, it was possible to cut the specimen without compromising the microscopic margin evaluation and the standard post-surgery clinical diagnostic workflow. A second method was used in cases where the largest dimension of the tumor was inferior to 1 cm. A different method was then required to ensure tissue cutting could be made without compromising the diagnosis.

For tumors larger than 1 cm, one or two samples were cut from different slices [Fig. 1(a)]. Those samples were selected by the pathologist (L.R.) based on visual inspection with the objective of having, whenever possible, *cancer*, *fat*, and *normal* breast tissue for each patient. The samples were placed on a black-coated aluminum sheet disposed on an expanded polystyrene (EPS) foam sheet. The orientation of the sample relative to the margins of the specimen (superior, inferior, posterior, and anterior) was noted on the EPS sheet, and a photograph was taken [Fig. 1(b)]. The EPS sheet was then placed under the imaging probe, and a white light measurement was taken before initiating the Raman imaging sequence [Fig. 1(c)].

For tumors smaller than 1 cm, the entire slice was left intact [Fig. 1(g)]. A slice selected by the pathologist after visual inspection of the specimen was placed on a black-coated aluminum sheet. The slice was then placed on an EPS sheet, and a note was made of the orientation of the margins. A custom 3D-printed guiding grid, for which all squares were the size of the system FOV, was created. The grid was then placed on top of the slice—without contact with the tissue—and a photograph was taken [Fig. 1(h)]. The pathologist indicated which square of the grid contained tumor tissue. The EPS sheet was placed under the imaging probe and aligned so the square area associated with the region of interest could be imaged. The selected square was identified, and the grid was carefully removed without moving the tissue slice. A white light measurement was taken before initiating the Raman imaging sequence [Fig. 1(i)].

For both methods, the laser power was set at maximum, resulting in ∼500  mW being delivered to the tissue surface over a line of width 350  μm. The line was scanned over the entire FOV, with each acquisition associated with three repeat measurements that were subsequently averaged to maximize the signal-to-noise ratio. The only exception was the fifth patient where the time allocated for acquisition was limited. Therefore, for that patient, the number of repeat measurements was set to one. Acquisition time was set at 10 s per line: 40 lines, for a total of 20 min when doing three repeat measurements. The exposure time was selected to respect the 1-h timeframe imposed by the pathologist after surgical excision to avoid tissue degradation that could compromise diagnostic accuracy.

**Fig. 2:**
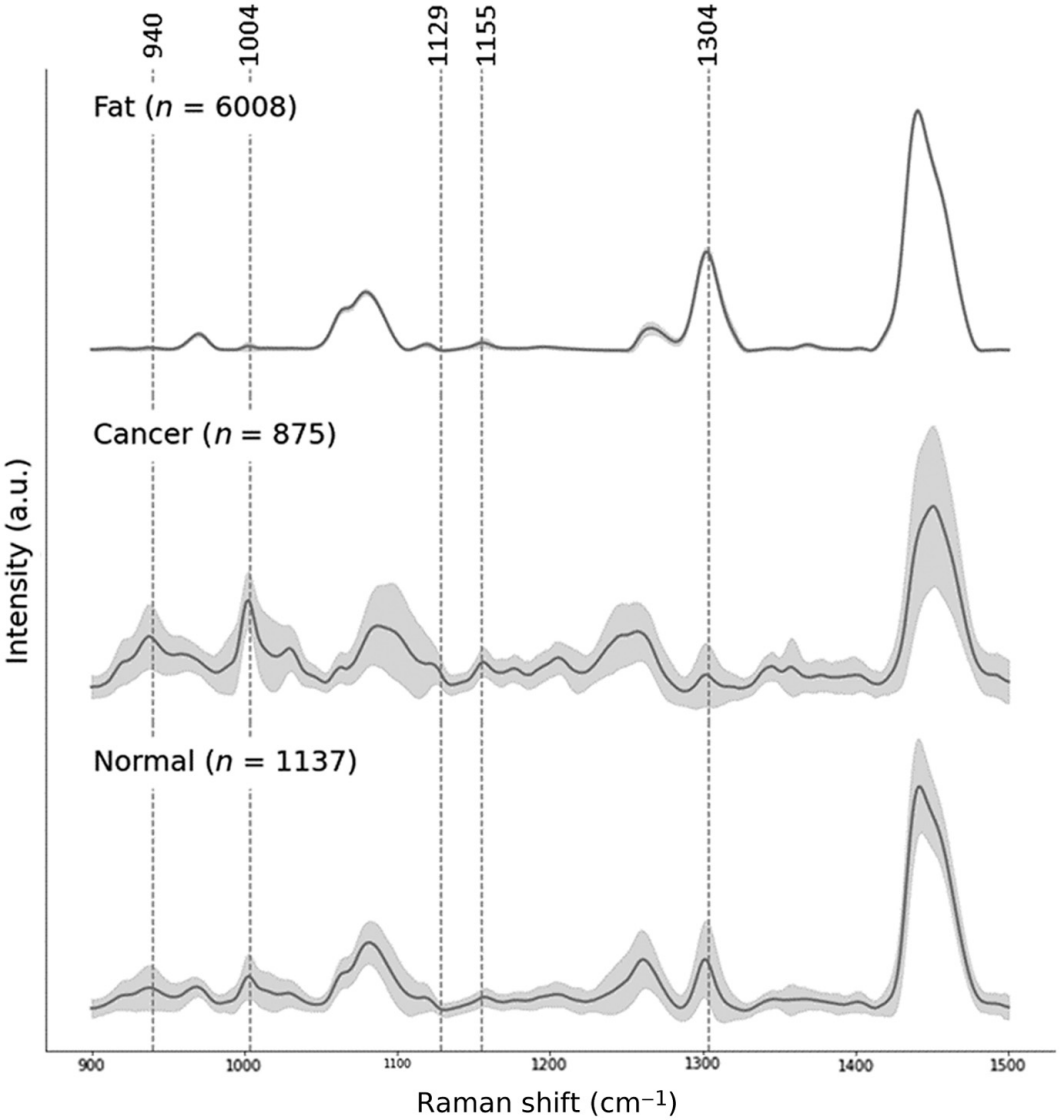
Average processed Raman spectra from breast tissue with standard deviation shown for each spectral bin. The spectra were classified into three categories: *fat* identified by model A, and *cancer* or *normal* predicted by model B after the exclusion of the *fat* spectra. The five spectral features used for classification (model A: 940, 1004, $1304\;{\mathrm{cm}}^{-1}$; model B: 940, 1004, 1129, $1155\;{\mathrm{cm}}^{-1}$) are identified by vertical lines.

After spectroscopic measurements, the orientation of the margins was marked with ink with different colors on each side of the sample. The samples were then placed into a pathology cassette [tumors larger than 1 cm, Fig. 1(e)]. The specimen slices were all pined to a new EPS sheet with the orientation of the margins written on it [(tumors smaller than 1 cm, Fig. 1(j)] as per standard of care. All tissues were then fixed in 10% neutral buffered formalin, embedded in paraffin, and sectioned into slides. They were then stained with hematoxylin and eosin (H&E) as per institutional standards, resulting in one stained image for each sample or one stained image for the entire slice if the tumor was smaller than 1 cm [Figs. 1(f) and 2(k)].

Digitized slides were reviewed by a pathologist (L.R.), who classified the tissue into three categories: (1) *cancer* (tumor cells, tumor stroma, or necrosis); (2) *normal*: either normal breast (connective tissue, stroma, fibroblast, collagen) or breast parenchyma (ducts and lobules); and (3) *fat* (adipose cells). The areas containing cancerous cells were annotated by the pathologist.

### 2.4 Registration with Optical Measurements and Histology Labels

Two three-step methodologies were developed to register spatially the histology images to the Raman hyperspectral images. For tumors larger than 1 cm, (1) the H&E-stained images were superposed to the photograph of the sample and rotated to ensure that the contours of the stained tissue sections matched as much as possible the contours seen in the photograph. The colored ink marking the margin orientation on the samples, as well as the irregularities associated with its shape, was used as geometrical landmarks guiding the spatial registration. (2) The white light image acquired with the system [Fig. 1(f)] was superposed to the picture of the sample with the margins annotated [Fig. 1(b)]. Again, margin orientation and visible structures, including blood vessels and regions with high adipose content, were used as tissue-based fiducial markers to ensure an accurate superposition of the white light image with the photograph of the specimen. (3) The histopathology image was then truncated to fit the size of the white light image and rotated 90 deg to compensate for the difference in orientation between white light images and Raman hyperspectral images. The resulting images were then comparable to the Raman hyperspectral image.

Similarly, three steps were followed for tumors smaller than 1 cm. (1) The H&E-stained images were superposed to the photograph of the specimen slice with the guidance grid [Fig. 1(h)] and rotated to ensure that the contours of the stained tissue sections matched as much as possible the contours seen in the photograph. (2) The white light image acquired with the system [Fig. 1(k)] was superposed to the specimen photograph and aligned with the corresponding square of the grid. (3) The histopathology image was then truncated to fit the size of the white light image and rotated 90 deg to compensate for the difference in orientation between white light images and Raman hyperspectral images.

### 2.5 Data Processing and Exclusion Criteria

The data processing steps preceding the application of the machine learning models led to the extraction of the inelastic scattering signature for each spectrum within the hyperspectral images. Background sources of signal non-specific to tissue Raman scattering, e.g., intrinsic fluorescence from tissue biomolecules,^49^ were isolated and removed from the spectra. Spectral pre-processing included the following steps: (1) median filter to each spectrum to eliminate cosmic ray events, (2) σ-axis (wavenumber shift) calibration based on a Raman spectrum acquired in acetaminophen, (3) normalization with a National Institute of Standards and Technology (NIST) Raman standard (SRM 2214) to correct for the instrument response, (4) removal of background signals using the custom algorithm BubbleFill,^50^ (5) removal of the background signal from the chromatic triplet of the system by subtracting an image acquired with nothing in focus, with a large integration time,^51^ and (6) standard normal variate (SNV) normalization. The five first columns of the hyperspectral images were excluded. The NIST standard being used to correct the instrument response was smaller than the FOV of the system; therefore, the correction could not be correctly performed for a small portion of the hyperspectral image.

A quantitative spectral quality factor (QF) metric (with a maximum value of 1) was computed for each spectrum of the image. It provided a statistical assessment of the likelihood the SNV-normalized signal was associated with tissue Raman peaks or stochastic noise.^52^ Spectra with a QF metric inferior to 0.5 were excluded from the images. The percentage of pixels associated with a QF factor inferior to 0.5 was computed for each image.

### 2.6 Machine Learning Workflow

A study was previously conducted involving 20 patients undergoing breast surgery.^46^ An RS point probe system was used to measure the spectral fingerprint of normal breast and cancer tissue *ex vivo.* Overall, 238 Raman spectra were acquired in that study consisting of 93 measurements associated with adipose tissue, 87 with cancer, and 58 with non-fat normal breast structures. These categories were labeled *fat*, *cancer*, and *normal*, respectively. This dataset was used to train classifiers using SVM to distinguish fat from normal structures, as well as to detect cancer out of non-fat normal breast tissue. The dimensional reduction was performed to ensure that the models used only a small number of biochemically interpretable spectral bands for classification. This also reduced the likelihood of overfitting and increased the model’s potential to generalize well to new data.

Two models trained on breast tissue spectra acquired with the point probe system were chosen for this study. The first model, model A, distinguished *fat* tissue from non-*fat* tissue (either *normal* or *cancer*) and was based on three spectral features centered at 940, 1004, and $1304\;{\mathrm{cm}}^{-1}$. The second model, model B, classified *cancer* versus *normal* tissue (excluding *fat* data) and required four spectral features centered 940, 1004, 1129, and $1155\;{\mathrm{cm}}^{-1}$. Those SVM models were directly applied to each of the spectra composing the hyperspectral Raman images acquired with the line-scanning system for eight breast specimens. Models A and B returned, for each spectrum, a posterior probability 0≤p≤1 that the spectrum belonged to a given class. To obtain predictions from these probabilities, a threshold of classification was needed. This threshold was chosen to optimize sensitivity and specificity during the previous study. A prediction was then obtained for the class of each spectrum (i.e., each pixel) composing the hyperspectral images.

The classification of the hyperspectral images was done in two steps. First, model A was applied to all spectra to identify *fat* spectra with the hyperspectral images. Then, model B was applied to the spectra identified as non-*fat* to classify them as either *cancer* or *normal*. The results of the classification were expressed through classification maps where each pixel corresponded to the tissue class predicted. The threshold of classification for model A to classify a spectrum as *fat* was increased to maximize sensitivity. This meant that less spectra were classified as *fat* since the spectra needed a higher probability p to be classified as *fat*. This limited the number of spectra falsely identified as *fat*. Increasing the threshold minimally affected the classification performance in the original point probe dataset (initially sensitivity/specificity was 96%/99% and, after increasing the threshold, it was 100%/95%). Adipose spectra not classified as *fat* were all classified as *normal*, which did not impact possible clinical applications as both *normal* and *fat* tissues are viewed as healthy tissue by pathologists and surgeons.

## 3 Results

### 3.1 Spectroscopic Measurements

The application of the Raman imaging protocol resulted in eight hyperspectral images from six patients with co-located histopathology analyses. A total of 11,520 spectra were acquired, with each spectrum corresponding to 1 pixel. After the exclusion of the spectra presenting a spectral QF inferior to 0.5, a total of 8020 spectra remained.

Classification sequentially used models A and B to split the dataset into three categories *fat* (n=6008 spectra), *normal* (n=1137), and *cancer* (n=875). Figure 2 presents the average processed spectra for each category and the standard deviation associated with each spectral bin. The spectral features used for tissue classification by the SVM models are highlighted in Fig. 2. The Standard deviation for *fat* spectra was smaller than *normal* and* cancer* due to a higher signal-to-noise ratio in *fat* spectra.

### 3.2 Machine Learning Models and Biomolecular Predictions

**Fig. 3:**
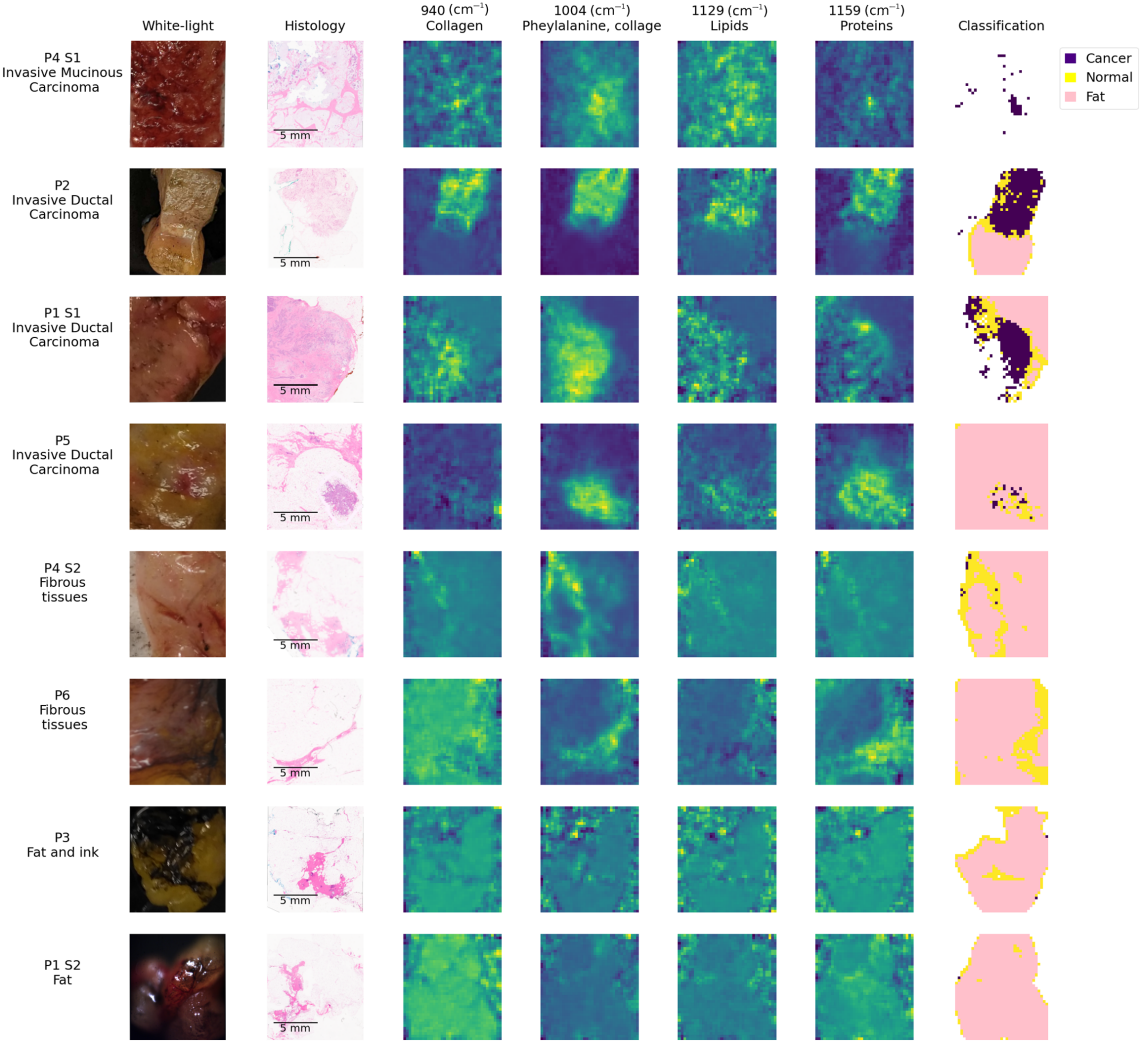
Imaging results for the eight samples considered in the study. Each line is for a different specimen, with the first column presenting an identification number and a label indicating tissue type. The second column presents a photograph of each specimen, and the third column shows the collocated H&E image used for microscopic analysis by a pathologist. The fourth, fifth, sixth, and seventh columns show inelastic scattering contrast for the Raman bands at 940, 1004, 1129, and $1159\;{\mathrm{cm}}^{-1}$, respectively. All images were normalized to their maximum value, and thus, all have a maximum value of 1 and a minimum value of 0. The eighth column presents the results of applying the machine learning models to each pixel image with the Raman system. Spectra that were classified as fat are in pink, spectra identified as normal or benign tissue are in yellow, and cancer predictions are represented by the color purple.

Figure 3 presents classification results for all eight samples considered in this study. Each row corresponded to a different sample that was labeled according to an identification number. An annotation was provided in Fig. 3 (first column) of the microscopic analysis results provided by a pathologist to establish the main tissue constituents. In cases where only adipose tissue could be detected by the pathologist, the label *fat* was used. The second column in Fig. 3 showed the photograph of the sample region that was imaged with the Raman imaging system, and the third column showed the corresponding histology image (stains: H&E) cropped to match the FOV of the Raman system. The fourth, fifth, sixth, and seventh columns showed the intensity maps for the spectral features 940, 1004, 1129, and $1159\;{\mathrm{cm}}^{-1}$, respectively. These bands corresponded to the spectral features included in model B trained to detect cancer from single-point RS measurements. The Raman band images were normalized to their maximum to ensure they were all represented on the same colormap to ensure relative intensities could be compared across all samples. The last column in Fig. 3 showed classification results for each spectrum as *fat*, *normal*, or *cancer*. The spectra with a QF below 0.5 were excluded from the classification maps.

Spectral images were acquired by summing data over three accumulations (i.e., three consecutive imaging sequences) to increase the signal-to-noise ratio. The same process as above was applied to spectral imaging data associated with only one accumulation, effectively reducing the image time by a factor of three (Fig. 5). The only specimen for which this was not done is P5 for which only one imaging sequence had been performed, i.e., no accumulation was possible.

The first four samples shown in Fig. 3 (P1-S1, P2, P4-S1, P5) contained cancer cells. The histology for P4-S1 showed that the sample was entirely composed of invasive mucinous carcinoma albeit with lower cellularity in the lower part of the image when compared with the upper area. The Raman models predicted that all pixels with QF > 0.5 were *cancer*. However, the spectral quality of the spectra in the image was very low for this specimen, and only 3% of all spectra remained after exclusion based on the spectral QF. The lower QFs associated with P4-S1 were associated with a larger fluorescence background—relative to the inelastic scattering signal—when compared with the other specimens in the study.

**Fig. 4:**
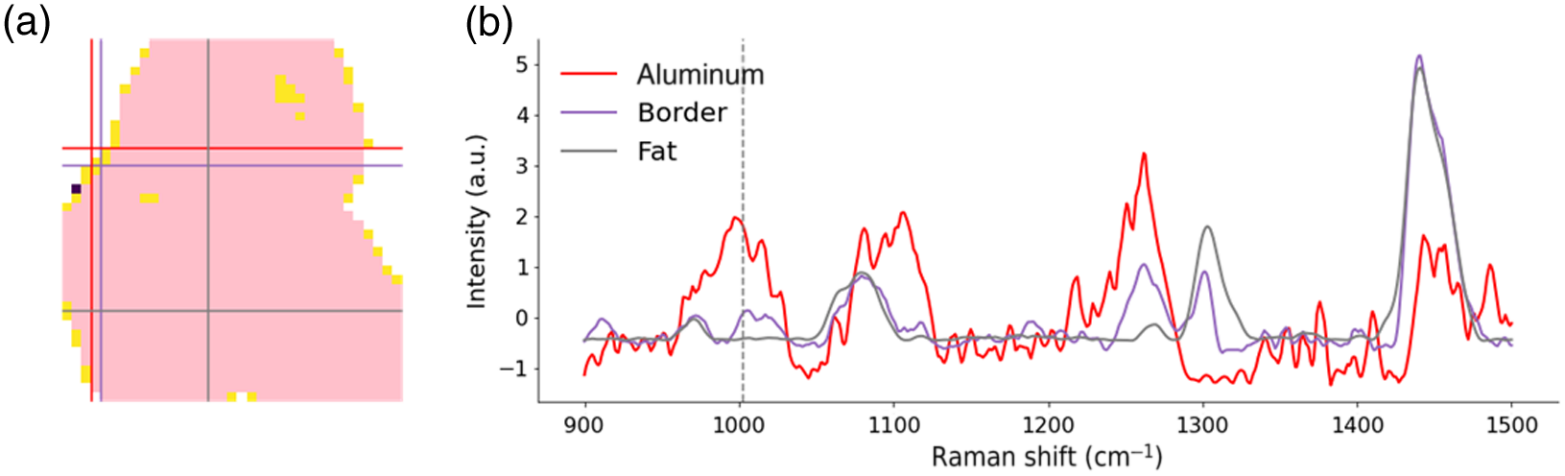
Classification results for specimen P1-S2 illustrating that the presence of non-tissue artifacts can confound machine learning model predictions: (a) classification map obtained when applying model A (trained to distinguish adipose tissue from either normal/benign or cancer tissue) followed by the application of model B (trained to distinguish normal/benign from cancer tissue) to pixels classified as non-adipose by model A. Pixels in pink are classified as *fat*, pixels in yellow as *normal*, and the only pixel classified as *cancer* is in purple. (b) Three representative Raman spectra are shown as follows: a spectrum from black-coated aluminum, a spectrum acquired at the border between the sample and the substrate, and a spectrum of adipose tissue. The location of each spectrum is indicated by a cross in panel (a) that has the same color as its corresponding spectrum in panel (b). A vertical line is used to indicate the spectral feature at $1004\;{\mathrm{cm}}^{-1}$ in panel (b).

The histology for P2 showed that half of the sample was constituted of invasive ductal carcinoma and the other half was adipose tissue. Model A identified adipose structures in a manner that was consistent with histology. Model B predicted that 74% of the remaining spectra belonged to the *cancer* category, providing an overall agreement with histology. As shown by the photograph of the sample, P2 was not large enough to fill the FOV of the imaging system. Spectra taken on regions where there was no biological sample (i.e., the black-coated aluminum substrate) were mostly excluded from classification due to low QF. However, spectra taken on the edge of the sample contained tissue signal and traces of black-coated aluminum Raman signal. In fact, classification results showed that spectra surrounding an adipose region were classified as *normal* instead of *fat*. This was due to the contamination of the adipose signal by the aluminum signal that contained an inelastic scattering signal around $1004\;{\mathrm{cm}}^{-1}$. The presence of an artifactual signal from the substrate confounded model predictions around the border of that specimen. This issue appeared on the boundary of all samples that were not large enough to fill the entire FOV of the system, i.e., P2, P1-S1, P4-S2, P3, and P1-S2. The effect of this signal contamination is illustrated in Fig. 4. This effect also resulted in the presence of individual pixels that were predicted as *cancer* around the periphery of specimens, including P4-S2.

For P1-S1, histology showed the sample contained invasive ductal carcinoma with high cellularity in the upper part of the image and adipose tissue in the upper right corner of the image. Model A correctly identified a *fat* region in the same region as the one shown by histology. Sixty-two percent of the remaining spectra were classified as *cancer* by model B. P5 contained a tumor of 3 mm size that was an invasive ductal carcinoma surrounded by adipose tissue and normal breast. Most of the spectra were classified as *fat* except for a small region at the bottom of the image that appeared to correspond to the tumor seen on histology, here slightly shifted to the left. After the exclusion of all *fat*-predicted spectra, model B classified 21% of the remaining spectra as *cancer* (34 spectra). The intensity map of the $1004\;{\mathrm{cm}}^{-1}$ band showed a content in the phenylalanine band for the entire tumor region that was like that associated with P4, P2, and P1-S1. The content in proteins, using the band at $1155\;{\mathrm{cm}}^{-1}$ as a surrogate, also appeared comparable to the other tumors.

The last four samples (P4-S2, P6, P3, P1-S2) contained no cancer cells. P4-S2 contained adipose and other normal breast tissue structures. Predictions of the models for that sample were consistent with histology, with most of the spectra being classified as *normal* or *fat*, and less than 1% of all pixels classified as *cancer*. The eight spectra on the edge of the sample classified as *cancer* contained a mix of *normal* tissue and aluminum signal, which resulted in a higher spectral feature around $1004\;{\mathrm{cm}}^{-1}$ (Fig. 4). The two other spectra classified as *cancer* had similar spectral features as *normal* spectra with a phenylalanine peak slightly higher. P6 contained mostly adipose tissue and a thin strip associated with other normal breast tissue structures. Model A classified 80% of all spectra as *fat*, with all other spectra being classified as *normal*. The histology for P3 showed mostly adipose and other normal breast tissue. The white light image of that specimen also showed residual black ink used for margin identification by the pathologist. This led to the exclusion of most of the spectra in the upper left part of the image based on low QF. Most of the remaining pixels for that sample were classified as *fat*. Histology for P1-S2 showed adipose and normal breast tissues. Most of the pixels in that image were classified as *fat*.

As previously mentioned, P2, P3, P4-S2, P3, and P1-S2 did not fill the entire FOV of the system, and Fig. 3 shows that the spectra at the border between the samples and the substrate were misclassified as *normal* or *cancer*. This was attributed to the contamination of the Raman signal by spectral features from the black-coated aluminum substrate, particularly around $1004\;{\mathrm{cm}}^{-1}$. Figure 4 presents the impact of the aluminum signal in the sample P1-S2. Figure 4(b) shows a spectrum of black-coated aluminum (excluded from the classification due to low QF), a spectrum from the edge of the sample, and a spectrum from adipose tissue. The location of each spectrum was indicated by a cross in the classification map [Fig. 4(a)]. Figure 4(b) shows an aluminum spectral feature around $1004\;{\mathrm{cm}}^{-1}$ that was present in the spectrum acquired on the edge of the sample, leading to the *normal* classification. Raman signal contamination by black ink was also observed in P3, and the spectra in the region with black ink were classified as *normal*. The samples P1-S2, P3, and P4-S2 also showed spectra classified as *cancer* on the edge of the samples where there was contamination by aluminum signal.

Table 3 reports that, for each Raman image, the percentage of pixels that had a QF inferior to 0.5, the percentage of all remaining pixels that were predicted as *fat*, and the percentage of pixels that were predicted as *cancer*. The latter metric was computed based only on the pixels that were predicted by model A not to belong to the *fat* category. Table 3 also includes the average posterior probability p (and its standard deviation) computed only based on the non-*fat* pixels. The latter metric could be used as a quantitative cancer burden marker, with larger values being associated with a more important burden. All specimens that contained cancer cells had an average p that was larger than 0.3, whereas all specimens that did not contain cancer had a value of 0.15 or inferior.

**Table 3:** Statistics for several metrics associated with specimen images acquired with the hyperspectral Raman images. Computed quantities include the percentage of pixels excluded from the analyses because they had a spectral QF inferior to 0.5, percentage of the remaining pixels that were classified as *fat* (model A), percentage of the pixels predicted as *cancer* by model B from non-fat measurements, and average and standard deviation (std) of the posterior probability value p that the non-*fat* pixels were predicted to belong to the *cancer* rather than the *normal* category (model B).

	Pathology	% excluded (QF <0.5)	% classified as *fat*	% classified as *cancer*	p [avg ± std]
P4 S1	Invasive mucinous carcinoma	97	0	100	0.98 ± 0.07
P2	Invasive ductal carcinoma	45	41	74	0.64 ± 0.28
P1 S1	Invasive ductal carcinoma	46	39	62	0.56 ± 0.32
P5	Invasive ductal carcinoma	0	91	22	0.31 ± 0.18
P4 S2	Fibrous tissue	13	79	4	0.15 ± 0.12
P6	Fibrous tissue	1	81	0	0.12 ± 0.07
P3	Adipose tissue	32	86	1	0.06 ± 0.09
P1 S2	Adipose tissue	17	96	2	0.06 ± 0.10

## 4 Discussion and Conclusion

This work demonstrated the potential of applying low-complexity machine learning models on hyperspectral Raman images acquired with a line-scanning macroscopic RS imaging system, to distinguish invasive breast cancer from healthy tissues. It also showed that to perform this task, it is feasible to use models trained using spectra acquired with a different system, i.e., a single-point RS system. Using two models developed from an *ex vivo* dataset acquired with the point probe, four tumors of different sizes and types were identified on the images from the hyperspectral imaging system. The overwhelming majority of image pixels associated with samples that were identified as healthy breast tissues by the pathologist were classified as non-*cancer*. However, not all classification results were consistent with histology results. Limitations included the potential misclassification of pixels where there were multiple contributors to the signal. For example, the aluminum substrate was shown to contaminate regions close to the edges of the specimens.

The accuracy of histopathology as a gold standard depended on the accuracy of the superposition between the Raman hyperspectral images and the histology maps. This was limited by the change in shape between the specimen photograph and the H&E-stained image of the corresponding tissue section. The samples were manipulated and rotated before being processed and stained, which sometimes made superposing the photograph with the H&E images difficult. This was particularly the case for samples composed of mostly adipose tissue since they did not retain their form as well as others.

Moreover, the H&E analysis was done on a tissue section of ∼5  μm thickness that was acquired before imaging with the Raman system. As a result, the surface on which the Raman measurements were made could be different than the surface from which histopathology information was derived. Another factor that can affect the spatial registration accuracy between histology and Raman-predicted classification is the diffuse nature of light propagation at near-infrared wavelengths. Because this phenomenon introduces a certain blurriness relating to the conveyed optical information, it limits the spatial resolution associated with the machine learning model predictions both axially and longitudinally. For example, diffusion enables light penetration into the tissue and leads to depth sampling up to a few hundred micrometers.^53^

Those limitations in spatial registration accuracy between histology and Raman-predicted maps did not allow a direct comparison of pixels from both image sets. This prevented an ROC analysis from being performed that would otherwise have allowed us to compute the cancer detection sensitivity and specificity of the new system. Rather, the overall ability of the system to detect cancer was assessed by computing the average posterior probability that pixels belonged to the cancer class across the whole image, after removal of the pixels that were predicted to be adipose tissue (Table 3).

Another limitation of the study was the small number of samples used to test the classification models developed with the single-point RS probe. There were two types of tumors included in the study, invasive ductal carcinoma and mucinous carcinoma. The latter was not present in the original training dataset and was nevertheless correctly classified as *cancer* in the present study. Invasive lobular carcinomas, included in the previous training dataset, were absent from the hyperspectral dataset, limiting the diversity of pathologies in this study.

Other practical limitations relate to the maximal permissible exposure (MPE) for skin as set by American National Standards Institute (ANSI) standards for research laboratory laser safety (ANSI Z136.1-2014 American National Standard for Safe Use of Lasers). For a total light exposure duration of 10 s and more, MPE for skin is $0.3\;W/{\mathrm{cm}}^{2}$. To respect this guideline, the laser power used with the Raman system should not be higher than 400 mW when computed for hazard evaluation (i.e., 3.5-mm diameter limiting aperture), whereas the power delivered at the surface of the tissue in this study was somewhat lower than 500 mW. However, it should be noted that those skin MPE values do not correspond to absolute thresholds beyond which tissue damage will necessarily result in breast tissue damage. For example, no morphological alterations were observed during histology analyses of the specimens in which RS measurements were made, suggesting that no heat-generated tissue damage was made. More work will be required to determine acceptable photodiagnostic exposure levels in breast tissue. They will likely be significantly larger than the current MPE values set for skin.

**Fig. 5:**
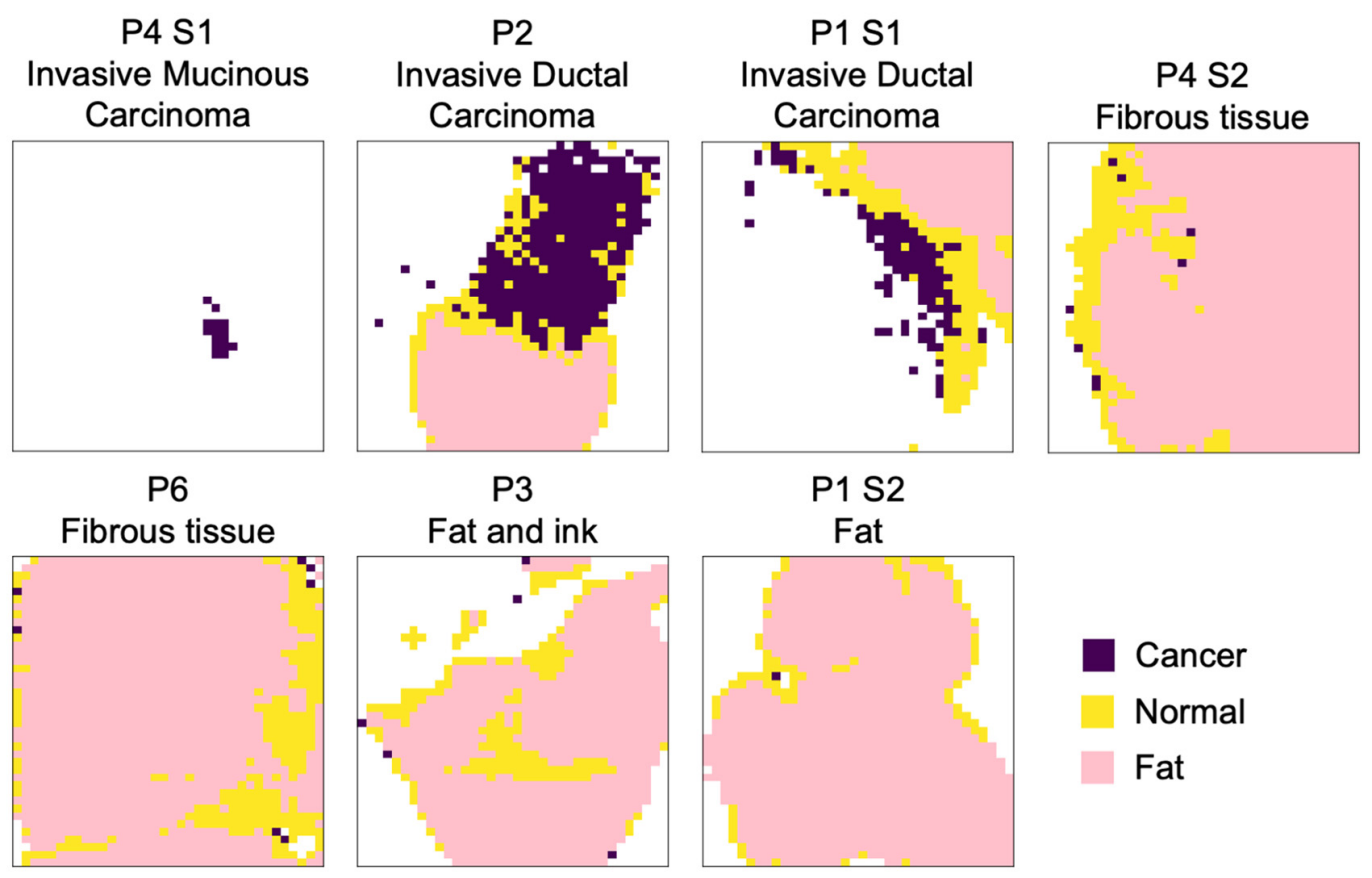
Imaging results for the samples considered in the study using only one out of three accumulations. P5 is not shown since only one accumulation was used for this specimen.

The total measurement time was 20 min per sample. This is too long for intraoperative clinical applications in which multiple measurements would be required to assess margins. However, measurement time could be lowered by taking one acquisition (i.e., one accumulation) instead of three, as was done for P5. This would bring the total imaging time to less than 7 min and allow for multiple measurements in approximately the same timeframe (20 to 30 min) as an intraoperative consultation by pathology.^54^ Raman-based classification maps were obtained for N=1 accumulation (Fig. 5). This led to results that associated approximately the same areas to *fat*, *normal*, and *cancer* categories, when compared with predictions associated with N=3 accumulations (Fig. 3).

The system could also be re-developed as a fiber-less system without the excitation and detection imaging bundles that were historically introduced to allow *in situ* and *in vivo* interrogation during surgery. This would limit light losses by at least a factor of 10, thereby potentially allowing more rapid imaging times by the same factor.^47^ Although outside of the scope of the work presented in this paper, such a system re-design would allow the development of a practical imaging workflow where all sides of a specimen could be imaged within a clinically compatible to fully assess whether residual cancer remains or not. Those improvements in light collection efficiency would allow for enhancing the imaging FOV, thereby allowing the development of workflows where whole lumpectomy specimens could be imaged. Future improvements in the technique should lead to a device and the development of clinical workflows allowing all sides of whole lumpectomy specimens—with their largest dimension typically nor larger than 5 cm—to be imaged in no more than 20 to 30 min. The latter figure was derived from the experience of the two surgeons involved in the study (S.M, F.T). The timeframe was established by weighing potential benefits to patients (diminishing risk of recurrence, improving cosmesis) against the impact of adding time to the procedure.

Other practical considerations include the requirement to develop clinical workflows whereby the surfaces of whole specimens are imaged prior to the use of pathology inks. This is because RS is a surface imaging modality sampling tissue at sub-millimeter depths. However, the line-scanning system was developed to allow variable software-controlled spatial offset between the excitation and detection lines to allow depth probing based on spatial offset RS.^48^ The use of this mode of operation is currently being evaluated as a means to image through a layer of ink, potentially alleviating the need to image the specimens prior to inking.

Another limitation of the current study is related to the fraction of measurements that were rejected based on the QF metric, ranging from 0% to 97% with an average of 31% (Table 3). Visual assessment of the rejected spectra showed that the vast majority were associated with measurements at the border of the specimens where tissue may have been thinner. The spectra associated with those measurements were all associated with Raman peaks from the aluminum substrate. This aspect was particularly dramatic for specimen P4-S1. In later work, this should be dealt with through the development of algorithms for automated spectral detection of substrate artifacts leading to the rejection of measurements.

This project showed the potential of line-scanning hyperspectral Raman imaging to provide intraoperative molecular analysis of *ex vivo* breast-conserving surgery specimens. This could be an important complement to gross margin evaluation and would offer a wide-field alternative to microscopic imaging methods such as frozen section techniques.

## Data Availability

The data and material information that support the findings of this study are available from the corresponding author upon reasonable request.
